# Mental Health Well-Being and Attitudes on Mental Health Disorders among Family Physicians during COVID-19 Pandemic: A Connection with Resilience and Healthy Lifestyle

**DOI:** 10.3390/jcm11020438

**Published:** 2022-01-15

**Authors:** Tina Vilovic, Josko Bozic, Sanja Zuzic Furlan, Marino Vilovic, Marko Kumric, Dinko Martinovic, Doris Rusic, Marko Rada, Marion Tomicic

**Affiliations:** 1 Department of Family Medicine, University of Split School of Medicine, 21000 Split, Croatia; tvilovic@mefst.hr (T.V.); sanja.zuzic@dz-sdz.hr (S.Z.F.); ravnatelj@dz-sdz.hr (M.R.); 2 Department of Family Medicine, Split-Dalmatia Health Center, 21000 Split, Croatia; 3 Department of Pathophysiology, University of Split School of Medicine, 21000 Split, Croatia; josko.bozic@mefst.hr (J.B.); marino.vilovic@mefst.hr (M.V.); marko.kumric@mefst.hr (M.K.); dinko.martinovic@mefst.hr (D.M.); 4 Department of Pharmacy, University of Split School of Medicine, 21000 Split, Croatia; doris.rusic@mefst.hr

**Keywords:** COVID-19, mental health, help-seeking, family physician, primary care, resilience, healthy lifestyle

## Abstract

Family physicians (FPs) are exposed to high amounts of stress, and could be susceptible to the development of mental health disorders (MHD), especially after the emergence of the COVID-19 pandemic. The aim of the current study was to assess MHD history, attitudes toward MHDs and stress-coping strategies in FPs. An additional goal was to estimate their comprehensive well-being and investigate connections with resilience and a healthy lifestyle. A total of 483 FPs submitted their responses via online survey. MHD attitudes were assessed with the according questionnaires, while burnout levels, healthy lifestyle, resilience, job and life satisfaction were estimated with validated scales. Results have shown that 32.5% of FPs disclosed positive MHD history, while 68.7% used professional help. Resilience and healthy lifestyle levels were significantly higher in MHD negative FPs (*p* < 0.001), while burnout levels were lower (*p* < 0.001). Moreover, healthy lifestyle (β = 0.03, *p* < 0.001) was an independent correlate of resilience, while healthy lifestyle (β = −0.35, *p* < 0.001, and resilience (β = −1.82, *p* < 0.001) were of burnout levels. Finally, resilience (OR = 0.387, *p* < 0.001) and healthy lifestyle (OR = 0.970, *p* = 0.021) were shown as independent predictors of positive MHD history status. Strong promotion and education of FP population regarding resilience and healthy lifestyle should be utilized in practice in order to alleviate the possibility of mental health disturbances and the according consequences.

## 1. Introduction

Several studies have shown that a large percentage of patients with mental health disorders (MHD) do not receive adequate treatment [1,2]. There could be multiple reasons for this phenomenon; however, the more important ones could be negative attitudes toward help-seeking behavior and various obstacles that emerge when confronted with knowledge or a subjective feeling of these specific medical diagnoses [2,3,4].

Physicians represent a population group known to have extremely stressful work, high rates of burnout, mental distress and suicide [5,6,7]. Additionally, the surrounding stigmatization of mental health diagnoses and barriers toward help-seeking could be even more pronounced when compared to other population groups, due to the negative attitudes and behaviors towards MHD, pessimism regarding treatment success, and the specific workplace culture [8]. It could be argued that these remarks could be even more pronounced in family physicians (FPs), as they represent cornerstones of the healthcare system, the point of trusted first contact for general population, and have numerous roles in society [9,10]. In addition, studies have shown that FPs are at higher risk of chronic stress and burnout than most of the other medical practitioners [11,12]. However, it is important to alleviate psychological distress, and to remove barriers to seeking professional help in FPs, due to possible consequences of untreated MHD, as well as observed connections with reduced patient content and treatment adherence, decreased productivity and increased rates of medical errors [13,14].

Rates of MHD were steadily rising over the last decade [15,16]; however, with the emergence of the coronavirus disease 2019 (COVID-19) pandemic, one of the main consequences of the re-structured lifestyle that followed was an additional spike in MHDs, especially in the population of healthcare workers that carry out a large organizational and management burden [17,18,19]. Therefore, in these demanding times, several countries recognized the problem and established various psychological support systems for this population [20].

Furthermore, to the best of our knowledge, only a few studies addressed help-seeking behaviors and attitudes toward MHDs in the physician population before the pandemic, and none in the primary medicine department [21,22]. Additionally, after the COVID-19 emergence, studies have shown low psychological help-seeking in several different population samples, including students, the general community, and healthcare workers [23,24,25]. In addition, investigations have shown the importance of appropriate coping with experienced stress, as some of the coping mechanisms can help us reduce stress in a manner that will promote positive psychological outcomes (“adaptive” coping), whereas some of them can lead to even more exacerbated stress in the long term, and promotion of poor mental health outcomes and increased psychopathology symptoms (“maladaptive coping”) [26,27]. However, there is a gap in the literature in help-seeking behaviors, stress-coping mechanisms, and experiences and attitudes toward MHDs from FPs, a population which has been substantially affected by the pandemic.

On the other hand, in the scientific literature, more emphasis is being put into investigating positive effects of resilience and healthy lifestyle on physicians’ mental health [28,29]. According to some authors, resilience can be considered as one of the most important elements to physicians’ well-being [29]. Even though there is no universal definition of resilience suggested in the literature [30], it could be broadly defined as a complex set of responses to traumatic and challenging situations that have fundamentals in three core components—insight, values, and self-care [31]. Further studies have shown that resilience could have some form of protective character regarding MHDs in physicians, however, there is still a scarcity of information of these connections in FP population, especially in COVID-19 pandemic era [11,32,33]. Furthermore, numerous evidence exists on the favorable effects of a healthy lifestyle on the individual’s well-being, which could include burnout as well [28,34]. However, there is limited information regarding the following of a healthy lifestyle in FPs, as well as connections to self-reported MHDs, resilience, and burnout. Finally, as previous studies have shown that burnout is closely connected to job satisfaction and various mental health disorders, and that resilience and a healthy lifestyle could be considered as a protective factor against experienced stress and burnout, we deem that it is important to explore probable connections between these constructs, with consideration of the history of mental health disorders in the FP population [28,29,31,33,34,35,36].

Therefore, the main goal of this study was to investigate the self-reported mental health disturbance history in a population of FPs, their attitudes toward MHD diagnosis, and stress coping strategies. An additional goal was to estimate current FPs’ mental health well-being through levels of burnout, job satisfaction, and satisfaction with life, and to investigate the connections with resilience and a healthy lifestyle.

## 2. Materials and Methods

### 2.1. Study Design and Participants

This cross-sectional study was conducted using a comprehensive online survey that was shared via the Google Forms^®^ platform. All current family physicians (physicians that work in family medicine practice) and family medicine residents (physicians in the current residency program in the field of family medicine) in the Republic of Croatia with minimally 2 years of working experience were eligible for inclusion. The link for the survey was shared via the official e-mail addresses of family medicine practices in the country, and via e-mails of family medicine associations in Croatia. Data were collected during the “third wave” of the COVID-19 pandemic in Croatia, between April and June of 2021. The main source of information of the family medicine practices in our country was the database available from the Croatian Institute for Health Insurance with 2337 listed practices with basic information. Afterwards, the link for the survey was sent to the acquired e-mail addresses that were collected after a rigorous search via several different resources (official online information, telephone calls, work contacts). In addition, contact was made with family medicine associations in Croatia, who forwarded the link to the survey through their channels. A total of 492 responses were collected; however, 9 of them were discarded due to reported FPs’ working experience being under 2 years. Hence, the overall response rate in this study was 21.0%, even though potential participants were reminded three time via e-mail and some of them via telephone calls.

All important information regarding this investigation was presented to invited FPs in the introduction of the survey and accompanying e-mail, while potential questions could be asked online. The identity of all included participants was secured with the according settings in Google forms^®^, while obtained informed consent was considered when the final submission of the answers was provided. The investigation was performed in accordance with the ethical standards of Declaration of Helsinki, and it was approved by the Ethics Committees of University of Split School of Medicine (No: 2181-198-03-04-21-0027) and Health Centre of the Split-Dalmatia County (No: 2181-149/01-21/01). Participation was voluntary, without compensation, while the asked questions did not include personal information.

### 2.2. Survey

Data were gathered using a comprehensive survey that was constructed at the Department of Family Medicine, University of Split School of Medicine after careful examination of the available literature by two FPs and one family medicine resident. Furthermore, each of the used statements and questions regarding experiences and attitudes on MHDs were carefully selected and adjusted after a detailed review of the similar studies [22,37,38,39], and after an additional consultation with a clinical psychologist.

Survey consisted of 3 main sections, with the first one exploring a total of 12 items that concentrated on general demographic data of the participants, as well as personal experience regarding MHDs. An MHD was defined as a problem with burnout, anxiety, depression, post-traumatic stress disorder (PTSD) or similar disorder that they consider to be in that group. Hence, gathered information included subjects’ gender, age, duration of work experience, patients in care, occupation, region of work and practice localization (urban area or rural area/islands). Furthermore, participants were asked whether they have family history of MHD, confirmed MHD diagnosis sometime in their careers, or if they were certain they have an MHD disorder; however, diagnosis was not officially confirmed. The last items from the first section were connected to the current pandemic, and they included information regarding self-assessed increased risk of COVID-19 adverse outcomes (due to older age or relevant chronic diseases), if they recovered from COVID-19, and if positive MHD status emerged during the pandemic.

The second section of the survey concentrated on the FP’s coping mechanisms with stress, as well as attitudes and personal experiences regarding help-seeking behaviors and MHD management. The participants were asked to express their attitudes on what they would be willing to do if confronted with MHD. Multiple answers could be selected from 6 different statements (“taking medication”, “going to psychotherapies”, “consultation with psychiatrist”, “trying to solve the problem alone”, “ignore the problem” and “talking to colleagues about it”). Furthermore, the second section included opinions on possible obstacles regarding seeking professional help. Again, multiple answers could be selected from the offered 5 statements (“no obstacles”, “not believing it would help”, “no time”, “fear of being incompetent for work”, “fear of colleagues’, patients’ or society stigmatization”). It should be noted that FPs with positive MHD history answered these questions about their personal experiences, while FPs without MHD history expressed their attitudes on the topic. Additionally, participants were asked what would be the best thing to acutely tackle their current mental health state, and they could choose answers from 5 different statements (“psychiatrist consultation”, help not needed”, “long vacation”, “self-help seminars” or “something else”).

Finally, the second section of the survey included analysis of FPs’ stress-coping mechanisms. They could choose how they usually behave when confronted with stress from 11 different items. Some of those items represent “adaptive” mechanisms, like “working out”, “spending time with family” or “communication with friends”, since studies have shown a positive connection between such constructs and favorable psychological outcomes [40,41,42]. In addition, some others can be considered as “maladaptive” mechanisms, since they are connected to negative mental health outcomes in time (“eating food”, “smoking”, “drinking alcohol”) [40,41,42].

The third part of the survey assessed various characteristics of FPs’ mental health well-being and lifestyle, including resilience, burnout, satisfaction with life and work, and the following of a healthy lifestyle.

Before the survey was forwarded to the entire FP population, it was pilot-tested on randomly chosen 16 family physicians and 5 family medicine residents for comprehensibility and duration assessment. All of the included participants answered to each of the items with ease, without reporting any understanding problems. In addition, they found that the survey duration time was acceptable as well (average 15 min). Hence, as there were no changes done in any part of the survey after pilot testing, the provided answers were included into the final analyses as well.

### 2.3. Mental Health Well-Being

For resilience assessment, two different questionnaires were used, that are based on different definitions of resilience in the scientific literature. Resilience can be defined as the ability of an individual to recover, or “bounce back” from a stressful situation [43,44,45]. Hence, Smith et al. introduced a Brief Resilience Scale (BRS), that consists of 6 different statements that could be answered through a 5-point Likert scale (ranged from “strongly disagree” to “strongly agree”) [45]. Results of points 1–5 were assigned considering the response, and the total score is formed as an arithmetic mean of the answers of all 6 items. Therefore, the total score on BRS ranged from 1.00–5.00, with higher scores representing a higher resilience trait. Based on a final result, FPs were put in three different categories, with the result of 1.00–2.99 representing “low resilience”, 3.00–4.30 “normal resilience”, and 4.31–5.00 “high resilience”. BRS is a widely used scale and already validated in different languages and population samples [46,47]. It was introduced and adapted to the Croatian language as well, with Cronbach’s alpha coefficient of 0.82, indicating acceptable internal consistency [48]. In our study population, the calculated Cronbach’s alpha coefficient was 0.84.

Furthermore, the second used questionnaire in our study that measures resilience is the Brief Resilience Coping Scale (BRCS), as resilience can be described as an ability to cope with stressful situations [49]. BRCS is translated and adapted to various languages and population samples as well, with acceptable measures of reliability [50,51]. It consists of 4 different statements to which participants expressed their agreement on 5-point Likert scale (ranging from “strongly disagree” to “strongly agree”). Points from 1 to 5 were assigned to each of the statements, with the total sum indicating the final score. Results from 4 to 13 represented “low resilient copers”, from 14 to 16 “medium resilient copers” and from 17 to 20 “high resilient copers”. Proper translation of BRCS was established with a back-translation technique by an English language expert. In our sample, Cronbach’s alpha coefficient was 0.79, indicating adequate internal consistency.

Burnout symptoms were evaluated with the Oldenburg Burnout Inventory (OBI), a validated and widely used questionnaire from Demerouti and Bakker [52]. This scale is conceptualized to measure two different dimensions of burnout—emotional exhaustion (OBI-E) and cognitive and somatic expressions of disengagement (OBI-D) [53]. Each of the subscales consists of 8 different statements to which subjects expressed agreement with a 4-point Likert scale (from “strongly disagree” to “strongly agree”). Each item was given 1–4 points, with consideration to items that were reversibly scored, with an increasing score indicating a higher level of burnout symptoms. Thus, the score for each of the subscales could range from 8 to 32 points. For the purposes of this study, as we did not find universal cut-off values that would determine different OBI groups, respondents were divided into tertile groups based on their score. OBI was adapted into the Croatian language as well, with good reliability results [48], while in our sample, the Cronbach’s alpha coefficient was 0.78 for the OBI-E subscale, and 0.85 for the OBI-D subscale, indicating good internal consistency as well.

The concept of satisfaction with life in our population was measured with the Satisfaction with Life Scale (SWLS), developed by Diener et al. [54]. It is one of the most commonly used and most reliable tools for this purpose [55]. It consists of 5 statements that describe different standards and expectations with life, to which participants subjectively respond using a 7-point Likert scale (from “strongly disagree” to “strongly agree”). Points from 1 to 7 were assigned to each answer, with higher scores indicating higher overall content with life. Furthermore, based on a final score, participants were put into 6 different groups: “extremely dissatisfied” (5–9 points), “dissatisfied” (10–14 points), “slightly below average” (15–19 points), “average” (20–24 points), “high score” (25–29 points) and “highly satisfied” (30–35 points). SWLS was translated using the back-translation technique, and it showed excellent internal consistency in our population sample (Cronbach’s alpha coefficient was 0.91).

Job satisfaction levels were assessed with the commonly used and validated Warr–Cook–Wall scale, with confirmed good psychometric properties [56,57]. The scale originally consists of 15 items; however, in the current paper, an abbreviated 10-item version was used, adapted specifically to be more appropriate for FPs [57]. Participants can describe their satisfaction with different aspects of work through the 7-point Likert scale (from “extremely dissatisfied” to “extremely satisfied”), with the total score ranging from 10 to 70 points, and higher scores indicating better overall job satisfaction. Proper translation of the scale was ensured with the back-translation technique, with Cronbach’s alpha coefficient of 0.86 indicating a good internal consistency measure.

Finally, physical, social and psychological aspects of a healthy lifestyle were assessed with the Fantastic Lifestyle Questionnaire (FLQ), an instrument developed originally by Wilson and Ciliska, and later adapted by the Canadian Society for Exercise Physiology to involve a more comprehensive view for each individual [58,59]. The questionnaire consists of a total of 25 questions that addresses the individual’s behavior in the last month, that are distributed into 9 different domains (F: family/friends; A: activity; N: nutrition; T: tobacco/toxins; A: alcohol; S: sleep/seatbelt/stress/safe sex; T: type of behavior; I: insight; C: career). Most of the items have 5 possible answers on a Likert scale, while two of them are dichotomous. After calculation, the final score can range from 0 to 100 points, with a higher score indicating healthier lifestyle behavior. Finally, according to the results, subjects were distributed into 5 different groups (0–34 points “needs improvement”; 35–54 points “fair”; 55–69 points “good”; 70–84 “very good” and 85–100 “excellent”). The questionnaire was translated with the back-translation technique, and has shown good reliability in other studies [60], as well as in ours, where the Cronbach’s alpha coefficient was 0.81.

### 2.4. Statistical Analysis

The appropriate sample size for this study was calculated via the online Surveymonkey^®^ calculator. The population that was eligible for this study consisted of 2337 FPs that were registered in the Republic of Croatia at the current time. The calculator has shown that the collected sample, with the 95% confidence interval and 5% error margin, should be at least 331 FPs. We succeeded in fulfilling these criteria, and acquired an even larger sample for adding further power to this investigation.

The MedCalc statistical program (version 19.1.2., MedCalc Software, Ostend, Belgium) was used for statistical analysis of the results. Whole numbers and percentages were used for categorical data presentation, with chi-squared test and Fisher’s exact test measuring statistical differences. D’Agostino–Pearson test was used for testing the normality of data distribution, and accordingly, continuous variables were presented as median and interquartile range, with Mann–Whitney U and Kruskall–Wallis test used to measure statistical differences. Furthermore, Spearman rank correlation coefficient was used to test the correlation between questionnaire scores and other relevant variables, while significant independent factors in association with burnout levels (according to total OBI score) and resilience (according to BRS score) were determined with multiple linear regression analysis. All the assumptions for using regression analysis were fulfilled, with enter selection algorithm used. Results were reported in the form of unstandardized beta coefficients (β), standard errors (SE), t values and *p*-values. Finally, in order to determine a relationship between selected independent variables and positive MHD history status in our participants, a multivariate logistic regression analysis with enter selection algorithm was used. The model was adjusted for age and gender, and inspected for the goodness of fit with the Hosmer Lemeshow test. Results were reported in the form of the adjusted odds ratios (OR), 95% confidence intervals and *p*-values. For this investigation, statistical significance was set at *p* < 0.05.

## 3. Results

### 3.1. Baseline Characteristics and Mental Health Experiences and Attitudes

The study included a total of 483 FPs (398 females and 85 males), from which 95 (19.7%) were family medicine residents. The median age of the population was 47.0 (33.0–58.0) years, while data were collected mostly from urban areas of the country (N = 329, 68.1%). The highest percentage of the population disclosed a number of patients between 1500 and 2000 (N = 199, 41.2%), while 125 (25.9%) subjects were recovered from COVID-19 to date (Table 1).

Self-assessment analysis revealed that a total of 157 (32.5%) FPs disclosed confirmed diagnosis or confident subjective perception of MHD, from which 77 (49.0%) were newly diagnosed from the start of the COVID-19 pandemic emergence. Furthermore, when compared to the population without MHD, FPs with a positive MHD history have a significantly higher percentage of population with positive family MHD experience (53.5 vs. 23.5%, *p* < 0.001), as well as those with increased personal risk of COVID-19 adverse outcomes (47.1 vs. 33.4%, *p* = 0.004). Detailed information regarding baseline characteristics according to MHD history can be seen in Table 1.

Further analysis showed that the majority of the population with MHD history (N = 108, 68.7%) chose to use some form of help, including medications, psychotherapies or psychiatrist consultations. Furthermore, experiences from MHD positive FPs significantly differed from MHD negative population attitudes, in terms of higher medication use (60.5 vs. 46.3%, *p* = 0.003), and a lower percentage of consultations with psychiatrist (29.9 vs. 44.2 %, *p* = 0.003). In addition, the highest percentage of the total investigated population (51.8%) disclosed trying to solve the problem alone (Table 2). Lastly, analysis of the items perceived as best for acute mental health management showed that the majority of FPs (64.6%) perceive a long vacation as something best currently needed, without significant differences according to the MHD history (*p* = 0.272) (Table 2). Subgroup analysis of the experiences on mental health management of FPs with MHD history according to MHD diagnostics (confirmed diagnosis vs. self-diagnosis) can be seen in Table S1, and according to the time of diagnosis (before COVID-19 pandemic vs. after COVID-19 pandemic) in Table S2.

**Figure 1:**
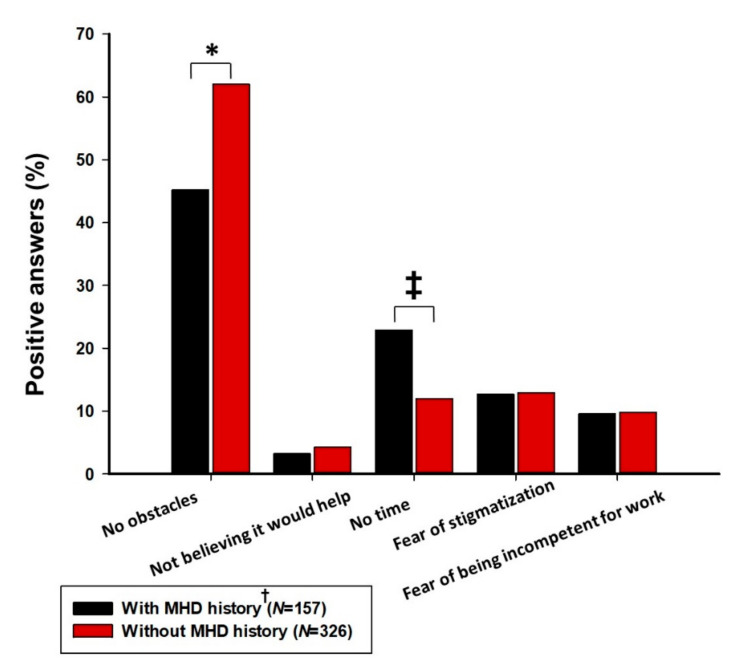
Experiences and attitudes regarding obstacles in seeking help for mental health disturbance according to the history of mental health disorders ^§^; MHD—mental health disorder; * chi-square test, *p* < 0.001; ^‡^ chi-square test, *p* = 0.002; ^†^ confirmed MHD diagnosis or positive subjective perception, ^§^ population with MHD history disclosed experiences, while population without MHD history disclosed attitudes.

When asked about obstacles to seeking professional help for MHD, the most common answer overall was “no obstacles” (N = 273, 56.5%), with a significantly higher percentage in population without MHD history (62.0 vs. 45.2%, *p* < 0.001) in comparison to MHD negative FPs. Other chosen items were “no time” (N = 75, 15.5%), with significantly higher prevalence in the MHD positive population (22.9 vs. 12.0%, *p* = 0.002) and “fear of stigmatization” (N = 62, 12.8%), without significant differences between the groups (*p* = 0.964) (Figure 1).

### 3.2. Stress-Coping Mechanisms and Current Mental Health Well-Being

Analysis of stress-coping mechanisms revealed that the most commonly used ones were “spending time with family” (N = 234, 48.4%), and “working out” (N = 224, 46.4%), while according to MHD history, both of them were used significantly more in MHD negative population (53.1 vs. 38.9%, *p* = 0.003 and 49.7 vs. 39.5%, *p* = 0.035, respectively). Furthermore, FPs with MHD history used significantly more mechanisms such as “drinking alcoholic drinks” (10.8 vs. 4.9%, *p* = 0.016), “watching television” (43.9 vs. 34.0%, *p* = 0.035) and “eating food” (36.3 vs. 19.9%, *p* < 0.001). Detailed information on used stress-coping mechanisms according to MHD history can be found in Table 3.

In this study, multiple questionnaires were used in order to acquire information on FPs’ mental health well-being. Analyses of the scales according to MHD history have shown that total scores that estimated resilience (BRS and BRCS), satisfaction with life and job (SWLS and WCW-JSS, respectively), and healthy lifestyle (FLQ) were higher in the MHD negative population, with robust significance levels (*p* < 0.001). Moreover, OBI scores that assessed burnout symptoms of exhaustion and disengagement, as well as cumulative score, were significantly lower in the same population of the MHD negative FPs, when compared to those with MHD history (*p* < 0.001) (Table 4). Subgroup analysis of mental health well-being questionnaire scores of FPs with MHD history according to MHD diagnostics can be seen in Table S1, and according to time of diagnosis in Table S2.

Further analysis revealed that BRS and FLQ scores showed significant positive correlation between them, as well as with the SWLS and WCW-JSS score (*p* < 0.001), while significant negative correlation was found with age, work experience and OBI scores (*p* < 0.001). In addition, a cumulative OBI score showed significant positive correlation with age (*p* = 0.038) and work experience (*p* = 0.019), while robust negative correlation was presented with SWLS and WCW-JSS scores (*p* < 0.001) (Table S3).

Statistical analyses of total OBI score when divided into tertile groups have revealed that the third tertile group had significantly more women (*p* = 0.006) and FPs with increased risk of COVID-19 adverse outcomes (*p* < 0.001) when compared to second and first tertile groups, as well as significantly less FPs with satisfied/highly satisfied life (29.9 vs. 50.3 vs. 77.6%, *p* < 0.001). In addition, analysis of selected coping mechanisms according to burnout tertile groups showed that the third tertile group had significantly more FPs that used mechanisms such as “eating food” (29.9 vs. 29.0 vs. 17.8%, *p* = 0.018) and “watching television” (44.2 vs. 38.1 vs. 30.5%, *p* = 0.036), while it had significantly less of those who chose item “spending time with family” (37.0 vs. 48.4 vs. 58.6%, *p* < 0.001) (Table 5).

**Figure 2:**
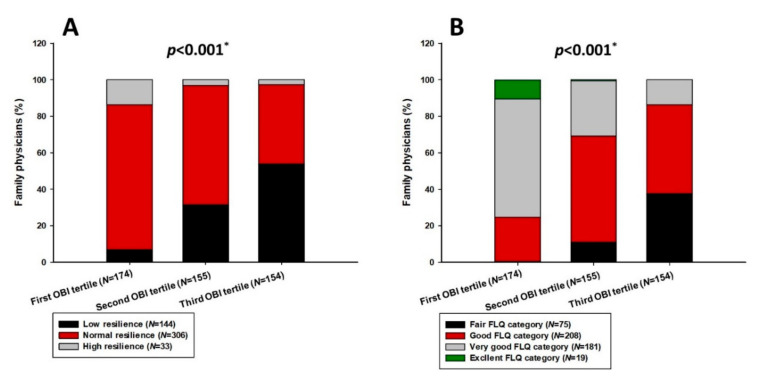
Resilience categories according to BRS scale (**A**) and healthy lifestyle categories according to FLQ scale (**B**) in OBI total score tertiles in study population; BRS—Brief Resilience Scale; FLQ—Fantastic Lifestyle Questionnaire; OBI—Oldenburg Burnout Inventory; * chi-square test.

Further analysis showed that the third OBI tertile group, in comparison with the other two, had significantly more participants with low resilience, according to the BRS scale (53.9 vs. 31.6 vs. 6.9%, *p* < 0.001) (Figure 2A), as well as significantly more of them in the fair lifestyle category (37.7 vs. 11.0 vs. 0.0%, *p* < 0.001) (Figure 2B).

Finally, the multiple linear regression model showed that the FLQ score (β = 0.03, SE = 0.003, *t*-value = 10.4, *p* < 0.001) was in significant association with the BRS score, set as dependent variable, when computed alongside baseline characteristics and SWLS score. Furthermore, a similar linear regression model that investigated independent predictors for burnout levels, with OBI cumulative score set as dependent variable, determined the FLQ score (β= −0.35, SE = 0.03, *t*-value= −11.4, *p* < 0.001), and BRS score (β= −2.12, SE = 0.44, *t*-value= −4.87, *p* < 0.001) to be the significant correlates.

Additionally, multivariate logistic regression analysis was performed in order to determine independent predictors of positive MHD history status. Model analysis showed BRS score (OR = 0.387, 95% CI = 0.261–0.574, *p* < 0.001) and FLQ score (OR = 0.970, 95% CI = 0.945–0.995, *p* = 0.021) to be significant predictors of MHDs in our population (Table S4).

## 4. Discussion

In this survey study, we investigated the prevalence of self-reported MHD history in the nation-wide population sample of FPs, as well as their attitudes and experiences toward MHDs. Additionally, we further addressed FPs’ well-being through assessment of burnout symptoms, job and life satisfaction, with analyzed connections to their resilience and healthy lifestyle following.

Results have shown than nearly one-third of the investigated population expressed a positive MHD history, from which 28% FPs had confirmed diagnosis. Furthermore, nearly 50% of MHD positive FPs developed these disturbances in the COVID-19 pandemic era. Moreover, the MHD positive group had a significantly larger percentage of FPs with an increased self-assessed risk of COVID-19 consequences when compared to the group without MHD history, that further attributes to the deleterious impact of the pandemic on mental health. Similar results were shown in a large cohort study of Australian frontline health workers, where 30% of the investigated population expressed a history of mental illness [61]. It can be argued that these results are in line with studies that confirmed the physician and FP population as susceptible to mental health disorders, especially in times of the COVID-19 pandemic [17,18,19]. However, according to the previous work on the similar FP population, there is a gap between percentages of anxiety, depression and PTSD symptoms based on validated questionnaires and the current self-report that expressed lower MHD prevalence [18]. This could be possible due to the different time period when the study has taken place, as well as possible self-perceived underestimation of low/moderate MHD symptoms that FPs could be used to and ignore.

Our results have shown that nearly 70% of FPs with MHD history actually had some form of professional help (medication, consultations, psychotherapies). This is a substantially larger percentage in comparison to the results of the studies conducted on medical workers in other countries during the pandemic [25,61,62]. These differences could be present due to several factors, including different cultural background, variability in formulated questions regarding help-seeking behavior, more recent timing in the pandemic in our study, differences in public health strategies, as well as the different population of healthcare workers. On the other hand, Muhamad Ramzi et al. conducted a study on a large cohort of physicians where obtained medical help for depression was around 60%, which is a similar result as in our study [63]. Although the current results imply that a favorable percentage of FPs actually took some form of mental health treatment, a large number of FPs still did not seek any kind of help. Additionally, the given percentage should be taken with caution due to the possibility of frequent self-medication in the physician population [64]. There is an interesting discrepancy between the attitudes in what MHD negative FPs think they would do if confronted with a disorder, and the actual history of actions of the MHD positive population. Taking medication was a more frequent answer in the MHD positive population, while consultation with a psychiatrist was a more common answer in the MHD negative population. It is possible that consultations are something that FPs would prefer to do in theory; however, due to the lack of available time, work overload or stigmatization, in the end, they put more effort into quicker solutions like medication.

Further analyses addressed attitudes on obstacles FPs face when seeking help for experienced MHD. The most commonly chosen answers were “no obstacles” and “no time”; however, again, there were significant differences on these attitudes according to the MHD history. FPs with a positive history had a lower answer rate on the “no obstacles” item, and a higher rate on the “no time” item. This is in line with the results of several other studies in which healthcare workers enclosed a lack of time as one of the most prominent reasons for not seeking mental health help [22,25]. These results are putting even more emphasis on the observation that FPs could change their attitudes when actually becoming unwell, and that they are indeed overwhelmed with work when they cannot find enough time to properly manage their mental health. These hypotheses are additionally supported with finding that most of the FPs believe a long vacation is the best thing to acutely tackle mental health state, regardless of MHD history.

Furthermore, the “fear of stigmatization” item was overall chosen in a somewhat lower percentage than expected (12.8%), when compared to other literature sources that addressed it as a major obstacle in seeking appropriate care [8,22,65]. Even though a systematic review by Clement et al., dating before the pandemic, associated a small to moderate cumulative negative effect of mental health stigma on help-seeking, it involved studies consisting of mixed population models [2]. Furthermore, it is possible that raising awareness about MHD in the pandemic time, encouraging the fight against stigmatization and severely deteriorating mental health well-being is facilitating favorable behaviors in the FP population, and reducing the effect of stigma. Nevertheless, mental health stigma still presents an important treatment obstacle in the physician population, and in these challenging times, it has never been more important to further raise awareness of mental health disturbances and to promote proper help seeking behaviors.

In order to assess the type of mechanisms by which FPs cope with daily stress, we offered them to choose from 11 different items, with the possible selection of multiple answers. Results have shown that the most commonly chosen mechanisms were working out (47%) and spending time with family (48%), which both can be considered as adaptive, positive coping mechanisms according to the available literature [40,41,42,66]. Moreover, these mechanisms were more represented in the group with a negative MHD history, and associated with lower burnout scores, which further emphasizes their commendatory, adaptive features. However, eating, watching television and drinking alcoholic drinks were moderately chosen mechanisms that were connected with a positive MHD history. Moreover, eating, watching television and smoking were represented more in higher burnout score groups. Studies have shown that the pandemic has had a negative effect on disordered eating behavior that could further be connected to increased psychological distress and job stress, which is in line with our observations [67]. When further comparing these results to other available similar studies, it can be observed that physical exercise is indeed one of most commonly used coping mechanisms in the healthcare population, while Smallwood et al. problematized increased alcohol use, which was connected to the history of poor mental health [38,61]. In addition, Wang et al. showed on a sample of Chinese physicians that those who had high perceived stress adopted more negative coping styles, which further led to higher levels of psychological distress [68]. According to all available information, it can be assumed that there is probable association between endured stress, MHDs and maladaptive coping skills. Healthy, positive coping mechanisms should be further promoted in the FP population in order to manage stress and possible MHD development more effectively [69].

Comprehensive evaluation of FPs’ well-being with validated scales revealed moderate resilience, satisfaction with life and healthy lifestyle characteristics. Further analysis showed that the group with positive MHD history had lower scores on resilience, healthy lifestyle and satisfaction with life and work, while burnout scores were higher. Furthermore, correlation analysis determined robust positive association between life and work satisfaction, healthy lifestyle and resilience. Moreover, healthy lifestyle following retained a significant connection with resilience after adjustment in the multiple regression model. On the contrary, the burnout score had a clear negative association with all of the other questionnaire scores, including resilience and healthy lifestyle, which was further confirmed in the group analysis and regression model.

When comparing acquired results to other studies that investigated effects of resilience on physicians’ burnout levels, similar associations were shown. In a study by Buck et al. conducted on family medicine residents and faculty members, regression models confirmed negative associations between depersonalization and emotional exhaustion with resilience, while corresponding results were shown in Australian general practice registrars, as well as the nurse population [33,70,71]. Similar conclusions were shown in the aforementioned studies despite the fact that burnout levels were measured via different scales. Considering the beneficial effects and importance of resilience, as well as the possibility of training it as an acquired skill [29], it is of utmost importance to learn more regarding factors that are positively influencing it. Moreover, literature has shown that physicians with higher resilience provide better quality of care for their patients, as well as reducing overall healthcare costs [72]. Hence, workplace intervention programs on healthcare workers have been described in the literature with some favorable findings; however, evidence is very limited and there is a need for more high-quality long-term studies [73,74].

It is interesting to notice healthy lifestyle as a factor between resilience and burnout, with significant connections to both. These results are in line with conclusions from recent investigations, which emphasized intensive healthy lifestyle as an important tool in burnout management and prevention, while it could be also seen as one of the fundamentals of resilience as a concept [28,31,34]. Our results further promote the idea of healthy lifestyle and resilience as key factors that could be used as a significant defense against work burnout in FP population. However, they could be strongly beneficial for overall mental health well-being as well, with results confirming both of them as significant independent predictors of MHD in a logistic regression model. It could be possible that individuals who demonstrate low resilience and live an unhealthy life are more susceptible in developing MHD; hence, these skills should be facilitated and worked on both personal and organizational levels. On the other hand, this may also be a two-way street, as FPs with MHDs may adopt an unhealthy lifestyle as a consequence of the mental disorder itself.

Several limitations of this study should be emphasized. With the cross-sectional investigation structure, causality between the acquired results cannot be assumed, while positive history of MHD in a family can be considered as a confounding factor. Furthermore, the history of MHD was based on a self-report by the investigated population, and not confirmed through official medical history records. In addition, specific medical diagnoses were not obtained and considered in the final analyses. Moreover, attitudes regarding specific mental health characteristics could be answered differently due to shame or perceived stigma. Hence, the number of the FPs with positive MHD history and results on MHD attitudes could be misinterpreted. However, as FPs are educated in recognizing relevant MHD symptoms and diseases, and with the anonymity guaranteed, it is safe to assume that answers were truthful and correct. Finally, much other collected information was based on a self-report as well, including the increased risk of COVID-19 adverse outcomes, practice localization and number of patients in practice, although we assume that FPs have enough knowledge to correctly answer these queries.

## 5. Conclusions

In conclusion, this study has shown that a relevant percentage of FPs experienced some form of mental health disturbances in their professional history, and that mental health severely deteriorated in the recent times of the COVID-19 pandemic. In addition, it is important to have in mind that a significant number of FPs still have obstacles that are preventing them from seeking adequate professional help. With the heavy burden they daily carry in the workplace, there is a strong need to continue raising awareness regarding MHD in this population, to encourage early help-seeking behavior, and to fight against stigmatization.

Furthermore, it is safe to assume that the FPs’ comprehensive well-being and a possibility of developing MHD is connected to each distinctive feature assessed in the present study. Hence, promotion of positive stress-coping abilities, as well as introduction and education regarding resilience and healthy lifestyle following should further be encouraged and investigated in order to improve overall health and to alleviate FPs from severe psychological distress. Finally, it is safe to assume that promotion of those characteristics can be expanded not only in the FP population, but to every patient in the clinical practice that is suffering from high work-related stress.

## Figures and Tables

**Table 1:** Baseline characteristics of study population according to mental health disorder medical history.

Parameter	with MHD History ^†^ (N = 157)	without MHD History (N = 326)	Total (N = 483)	*p* *
Women	133 (84.7)	265 (81.3)	398 (82.4)	0.355
Age (years)	48.0 (33.0–58.0)	46.5 (33.0–58.0)	47.0 (33.0–58.0)	0.992 ^‡^
Work experience (years)	20.0 (5.0–30.0)	13.0 (6.0–28.0)	15.0 (6.0–30.0)	0.842 ^‡^
Occupation				
Family physician	132 (84.1)	256 (78.5)	388 (80.3)	0.151
Family medicine resident	25 (15.9)	70 (21.5)	95 (19.7)	
Region of work				
Adriatic region	62 (39.5)	144 (44.2)	206 (42.7)	0.579
North-West region	38 (24.2)	77 (23.6)	115 (23.8)	
Central and East region	57 (36.3)	105 (32.2)	162 (33.5)	
Practice localization				
Urban area	104 (66.2)	225 (69.0)	329 (68.1)	0.540
Rural area/islands	53 (33.8)	101 (31.0)	154 (33.8)	
Patients in practice				
<1000	7 (4.5)	21 (6.4)	28 (5.8)	0.311
1000–1500	40 (25.5)	90 (27.6)	130 (26.9)	
1500–2000	73 (46.5)	126 (38.7)	199 (41.2)	
>2000	30 (19.1)	80 (24.5)	110 (22.8)	
Not answered	7 (4.5)	9 (2.8)	16 (3.3)	
Family history of MHD	84 (53.5)	77 (23.6)	161 (33.3)	<0.001
Increased COVID-19 risk ^§^	74 (47.1)	109 (33.4)	183 (37.9)	0.004
Recovered from COVID-19	38 (24.2)	87 (26.7)	125 (25.9)	0.559

Data are presented as N (%) or median (interquartile range); MHD—mental health disorder; COVID-19—coronavirus disease 2019; * chi-square test; ^‡^ Mann–Whitney U test; ^†^ confirmed MHD diagnosis or positive subjective perception; ^§^ increased self-assessed risk from COVID-19 adverse outcomes.

**Table 2:** Experiences and attitudes on mental health management according to MHD history.

Parameter	with MHD History ^†^ (N = 157)	without MHD History (N = 326)	Total (N = 483)	*p* *
**Actions regarding knowledge of MHD ^‡^**				
Taking medication	95 (60.5)	151 (46.3)	246 (50.9)	0.003
Going to psychotherapies	41 (26.1)	108 (33.1)	149 (30.8)	0.118
Consultation with psychiatrist	47 (29.9)	144 (44.2)	191 (39.5)	0.003
Trying to solve the problem alone	85 (54.1)	165 (50.6)	250 (51.8)	0.468
Ignore the problem	7 (4.5)	2 (0.6)	9 (1.9)	0.006
Talking with colleagues about it	0 (0.0)	5 (1.5)	5 (1.0)	0.179
**Best thing to acutely tackle mental health state**				
Psychiatrist consultation	38 (24.2)	18 (5.5)	56 (11.6)	<0.001
Help not needed	9 (5.7)	69 (21.2)	78 (16.1)	<0.001
Long vacation	96 (61.1)	216 (66.3)	312 (64.6)	0.272
Self-help seminars	27 (17.2)	38 (11.7)	65 (13.5)	0.095
Something else	9 (5.7)	12 (3.7)	21 (4.3)	0.301

Data are presented as N (%); MHD: mental health disorder; * chi-square test or Fisher’s exact test; ^†^ confirmed MHD diagnosis or positive subjective perception; ^‡^ population with MHD history disclosed experiences, while population without MHD history disclosed attitudes.

**Table 3:** Coping mechanisms for stress relief according to the history of mental health disorders in study population (N = 483).

Parameter	with MHD History ^†^ (N = 157)	without MHD History (N = 326)	Total (N = 483)	*p* *
Working out	62 (39.5)	162 (49.7)	224 (46.4)	0.035
Listening to music	61 (38.9)	136 (41.7)	197 (40.8)	0.549
Smoking	21 (13.4)	35 (10.7)	56 (11.6)	0.396
Drinking alcoholic drinks	17 (10.8)	16 (4.9)	31 (6.4)	0.016
Spending time with family	61 (38.9)	173 (53.1)	234 (48.4)	0.003
Working on business projects	7 (4.5)	18 (5.5)	25 (5.2)	0.621
Religious/Spiritual activities	22 (14.0)	44 (13.5)	66 (13.7)	0.877
Reading	47 (29.9)	108 (33.1)	155 (32.1)	0.482
Watching television	69 (43.9)	111 (34.0)	180 (37.3)	0.035
Communication with friends	40 (25.5)	78 (23.9)	118 (24.4)	0.710
Eating food	57 (36.3)	65 (19.9)	122 (25.3)	<0.001

Data are presented as N (%), MHD- mental health disorder, * chi-square test; ^†^ confirmed MHD diagnosis or positive subjective perception.

**Table 4:** Total scores of used questionnaires investigating burnout levels, resilience, satisfaction with life and job, and healthy lifestyle in family physicians according to the history of mental health disorders.

Parameter	with MHD History ^†^ (N = 157)	without MHD History (N = 326)	Total (N = 483)	*p* *
BRCS score	15.0 (12.0–16.0)	16.0 (14.0–17.0)	15.0 (14.0–17.0)	<0.001
BRS score	2.83 (2.33–3.5)	3.33 (3.0–3.83)	3.33 (2.83–3.79)	<0.001
FLQ score	61.0 (51.0–71.0)	68.0 (62.0–77.0)	68.0 (59.0–75.0)	<0.001
OBI exhaustion	23.0 (20.0–26.0)	21.0 (18.0–24.0)	21.0 (19.0–25.0)	<0.001
OBI disengagement	21.0 (18.0–23.0)	19.0 (17.0–21.0)	19.0 (17.0–21.0)	<0.001
OBI total	44.0 (40.0–49.2)	40.0 (35.0–44.0)	41.0 (36.0–46.0)	<0.001
SWLS score	22.0 (16.0–26.0)	26.0 (22.0–30.0)	25.0 (19.0–29.0)	<0.001
WCW-JSS score	43.0 (36.7–50.0)	48.0 (40.0–55.0)	47.0 (39.2–54.0)	<0.001

Data are presented as median (interquartile range); MHD—mental health disorder; BRCS—Brief Resilient Coping Scale; BRS—Brief Resilience Scale; FLQ—Fantastic Lifestyle Questionnaire; OBI—Oldenburg Burnout Inventory; SWLS—Satisfaction with Life Scale; WCW-JSS—Warr-Cook-Wall Job Satisfaction Scale; * Mann–Whitney U test; ^†^ confirmed MHD diagnosis or positive subjective perception.

**Table 5:** OBI total score tertiles according to various relevant parameters in study population.

Parameter	1. Tertile (N = 174)	2. Tertile (N = 155)	3. Tertile (N = 154)	*p* *
Age (years)	41.0 (33.0–57.0)	46.0 (33.0–58.0)	50.0 (39.0–58.0)	0.041 ^‡^
Women	131 (75.3)	131 (84.5)	136 (88.3)	0.006
With MHD history ^†^	35 (20.1)	49 (31.6)	73 (47.4)	<0.001
Increased COVID-19 risk ^§^	43 (24.7)	66 (42.6)	74 (48.1)	<0.001
Practice localization				
Urban area	119 (68.4)	95 (61.3)	115 (74.7)	0.041
Rural area/islands	55 (31.6)	60 (38.7)	39 (25.3)	
Occupation				
Family physician	136 (78.2)	115 (74.2)	137 (89.0)	0.003
Family medicine resident	38 (21.8)	40 (25.8)	17 (11.0)	
BRCS categories				
Low resilient coping	17 (9.8)	36 (23.2)	55 (35.7)	<0.001
Medium resilient coping	83 (47.7)	88 (56.8)	82 (53.2)	
High resilient coping	74 (42.5)	31 (20.0)	17 (11.0)	
SWLS categories				
Dissatisfied/Extremely dissatisfied	8 (4.6)	16 (10.3)	38 (24.7)	<0.001
Average/Slightly below average	31 (17.8)	61 (39.4)	70 (45.5)	
Satisfied/Highly satisfied	135 (77.6)	78 (50.3)	46 (29.9)	
Selected coping mechanisms				
Eating food	31 (17.8)	45 (29.0)	46 (29.9)	0.018
Smoking	12 (6.9)	21 (13.5)	23 (14.9)	0.049
Watching television	53 (30.5)	59 (38.1)	68 (44.2)	0.036
Spending time with family	102 (58.6)	75 (48.4)	57 (37.0)	<0.001
Working out	90 (51.7)	64 (41.3)	72 (46.8)	0.166

Data are presented as N (%) and median (IQR) where appropriate; COVID-19: coronavirus disease 2019; MHD: mental health disorder; * chi-square test; ^‡^ Kruskall–Wallis test; ^†^ confirmed MHD diagnosis or positive subjective perception; ^§^ increased self-assessed risk from COVID-19 adverse outcomes.

## Data Availability

The data presented in this study are available on request from the corresponding author. The data are not publicly available due to ethical restrictions.

## Supplementary Materials

The following are available online at https://www.mdpi.com/article/10.3390/jcm11020438/s1, Table S1: Experiences on mental health management and mental health well-being questionnaire scores according to diagnostics of MHD in a group of participants with positive MHD history (N = 157), Table S2: Experiences on mental health management and mental health well-being questionnaire scores according to time of diagnosis in a group of participants with positive MHD history (N = 157), Table S3: Correlation of resilience, burnout and healthy lifestyle questionnaire scores with other relevant parameters in the total study population (N = 483), Table S4: Multivariate logistic regression analysis of independent predictors for positive mental health disorder history status
