# Supplementary material for: Mental Health Well-Being and Attitudes on Mental Health Disorders among Family Physicians during COVID-19 Pandemic: A Connection with Resilience and Healthy Lifestyle
Source: J Clin Med. 2022 Jan 15;11(2):438. doi: 10.3390/jcm11020438 (PMC8778288; doi:10.3390/jcm11020438)
Supplement: Supplementary file 1 [file jcm-11-00438-s001.zip › jcm-1518908-supplementary.pdf]

Supplementary material

Table S1. Experiences on mental health management and mental health well-being questionnaire scores according to diagnostics of MHD in a group of participants with positive MHD history (N=157)

Parameter	Confirmed MHD history (N=44)	Positive MHD perception (N=113)	Total (N=157)	p^*
Actions regarding knowledge of MHD				
Taking medication	33 (75.0)	62 (54.9)	95 (60.5)	0.021
Going to psychotherapies	20 (45.5)	21 (18.6)	41 (26.1)	<0.001
Consultation with psychiatrist	23 (52.3)	24 (21.2)	47 (29.9)	<0.001
Trying to solve the problem alone	16 (36.4)	69 (61.1)	85 (54.1)	0.005
Ignore the problem	2 (4.5)	5 (4.4)	7 (4.5)	0.998
Best thing to acutely tackle mental health state				
Psychiatrist consultation	21 (47.7)	17 (15.0)	38 (24.2)	<0.001
Help not needed	4 (9.1)	5 (4.4)	9 (5.7)	0.268
Long vacation	17 (38.6)	79 (69.9)	96 (61.1)	<0.001
Self-help seminars	3 (6.8)	24 (21.2)	27 (17.2)	0.034
Something else	2 (4.5)	7 (6.2)	9 (5.7)	0.998
Mental health well-being questionnaire scores				
BRCS score	16.0 (12.0-16.0)	15.0 (12.0-16.0)	15.0 (12.0-16.0)	0.194†
BRS score	2.75 (2.41-3.5)	2.83 (2.33-3.67)	2.83 (2.33-3.5)	0.683†
FLQ score	68.0 (56.0-74.0)	60.0 (50.7-69.2)	61.0 (51.0-71.0)	0.067†
OBI exhaustion	23.0 (18.5-26.0)	24.0 (21.0-26.0)	23.0 (20.0-26.0)	0.160†
OBI disengagement	20.0 (18.0-22.5)	21.0 (19.0-24.0)	21.0 (18.0-23.0)	0.190†
OBI total	44.5 (37.5-47.0)	44.0 (40.0-50.0)	44.0 (40.0-49.2)	0.165†
SWLS score	20.5 (17.5-25.5)	22.0 (15.7-26.0)	22.0 (16.0-26.0)	0.975†
WCW-JSS score	47.0 (39.0-50.0)	43.0 (36.0-50.0)	43.0 (36.7-50.0)	0.581†

Data are presented as N (%) and median (interquartile range)

MHD- mental health disorder; BRCS- Brief Resilient Coping Scale; BRS- Brief Resilience Scale; FLQ- Fantastic Lifestyle Questionnaire; OBI- Oldenburg Burnout Inventory; SWLS- Satisfaction with Life Scale; WCW-JSS- Warr-Cook-Wall Job Satisfaction Scale

* chi-square test or Fisher's exact test

† Mann-Whitney U test

Table S2. Experiences on mental health management and mental health well-being questionnaire scores according to time of diagnosis in a group of participants with positive MHD history (N=157)

Parameter	During COVID-19 pandemic (N=77)	Before COVID-19 pandemic (N=80)	Total (N=157)	p^*
Actions regarding knowledge of MHD				
Taking medication	51 (66.2)	44 (55.0)	95 (60.5)	0.151
Going to psychotherapies	12 (15.6)	29 (36.2)	41 (26.1)	0.003
Consultation with psychiatrist	24 (31.2)	23 (28.7)	47 (29.9)	0.742
Trying to solve the problem alone	48 (62.3)	37 (46.2)	85 (54.1)	0.043
Ignore the problem	4 (5.2)	3 (3.7)	7 (4.5)	0.716
Best thing to acutely tackle mental health state				
Psychiatrist consultation	21 (27.3)	17 (21.2)	38 (24.2)	0.380
Help not needed	0 (0.0)	9 (11.3)	9 (5.7)	0.002

Long vacation	57 (74.0)	39 (48.7)	96 (61.1)	0.001
Self-help seminars	16 (20.8)	11 (13.8)	27 (17.2)	0.244
Something else	2 (2.6)	7 (8.8)	9 (5.7)	0.168
Mental health well-being questionnaire scores				
BRCS score	15.0 (13.7-16.0)	15.0 (12.0-16.0)	15.0 (12.0-16.0)	0.424†
BRS score	2.83 (2.33-3.5)	2.83 (2.41-3.67)	2.83 (2.33-3.5)	0.593†
FLQ score	61.0 (51.0-68.0)	61.0 (52.7-76.5)	61.0 (51.0-71.0)	0.042†
OBI exhaustion	25.0 (23.0-27.0)	21.5 (19.0-25.0)	23.0 (20.0-26.0)	<0.001†
OBI disengagement	21.0 (20.0-24.0)	20.0 (17.0-22.0)	21.0 (18.0-23.0)	<0.001†
OBI total	46.0 (42.7-52.0)	40.0 (36.0-47.0)	44.0 (40.0-49.2)	<0.001†
SWLS score	22.0 (15.7-26.2)	21.5 (17.0-26.0)	22.0 (16.0-26.0)	0.679†
WCW-JSS score	41.0 (35.7-49.0)	47.0 (39.0-52.0)	43.0 (36.7-50.0)	0.013†

Data are presented as N (%) and median (interquartile range)
MHD- mental health disorder; BRCS- Brief Resilient Coping Scale; BRS- Brief Resilience Scale; FLQ- Fantastic Lifestyle Questionnaire; OBI- Oldenburg Burnout Inventory; SWLS- Satisfaction with Life Scale; WCW-JSS- Warr-Cook-Wall Job Satisfaction Scale
* chi-square test or Fisher's exact test
† Mann-Whitney U test

Table S3. Correlation of resilience, burnout and healthy lifestyle questionnaire scores with other relevant parameters in total study population (N=483)

Parameter	BRS<br>*r* (*p**)	OBI total score<br>*r* (*p**)	FLQ<br>*r* (*p**)
Age (years)	-0.157 (<0.001)	0.094 (0.038)	-0.124 (0.006)
Work experience (years)	-0.173 (<0.001)	0.106 (0.019)	-0.105 (0.021)
BRCS score	0.390 (<0.001)	-0.393 (<0.001)	0.473 (<0.001)
BRS score	-	-0.503 (<0.001)	0.532 (<0.001)
FLQ score	0.532 (<0.001)	-0.656 (<0.001)	-
OBI-exhaustion	-0.511 (<0.001)	0.937 (<0.001)	-0.633 (<0.001)
OBI-disengagement	-0.404 (<0.001)	0.899 (<0.001)	-0.572 (<0.001)
OBI total score	-0.503 (<0.001)	-	-0.656 (<0.001)
SWLS score	0.394 (<0.001)	-0.437 (<0.001)	0.545 (<0.001)
WCW-JSS score	0.316 (<0.001)	-0.538 (<0.001)	0.484 (<0.001)

BRCS- Brief Resilient Coping Scale; BRS- Brief Resilience Scale; FLQ- Fantastic Lifestyle Questionnaire; OBI- Oldenburg Burnout Inventory; SWLS- Satisfaction with Life Scale; WCW-JSS- Warr-Cook-Wall Job Satisfaction Scale
* Spearman rank correlation

Table S4. Multivariate logistic regression analysis of independent predictors for positive mental health disorder history status

Variables	OR	95% CI	*p*
Age (years)	0.987	0.971-1.004	0.120
Gender (male vs. female)	0.890	0.499-1.585	0.693
BRS score	0.387	0.261-0.574	<0.001
FLQ score	0.970	0.945-0.995	0.021

OBI total score	1.014	0.976-1.053	0.455

BRS- Brief Resilience Scale; FLQ- Fantastic Lifestyle Questionnaire; OBI- Oldenburg Burnout Inventory; 95% CI- 95% confidence interval; OR- adjusted odds ratio
